# Food in War-Time

**Published:** 1915-04-17

**Authors:** 


### FOOD IN WAR-TIME.

In this time of anxiety and stress the British public has little reason to complain of any failure in the supply of advice and advisers. Indeed, the flow is overwhelming, and it is hardly possible to avoid the torrent. We will confine ourselves to those fashions of advice which deal with medical and physiological questions. If in so doing we appear to swell a chorus already sufficiently full-bodied, our critics may find in our action a chance for a reasonable revenge.

One of the most attractive of the organised efforts to lead public opinion was a recent discussion—arranged with due pomp and circumstance—on "Our Food during War." The subject was obviously appropriate to the hour, and a wider appeal cannot well be imagined. It is not difficult to picture the numerous patriotic households in which instruction was anticipated with the keenest interest. The prospect of learning how to combine economy and efficiency with love of country and sound physiological principles is an alluring one, and must have filled many minds with confident expectation and hope. The only shadow of anxiety which may have weighed upon the organisers was perhaps the thought that they might, all unintentionally, be contributing to the relief of the enemy. Unless rumour is even more ill-informed than usual, food problems are more acute with the foe than with ourselves, and it would be a dreadful thing to think that good advice intended for home consumption might possibly be used to ease the anxieties of Teutonic organisers. However, all great enterprises involve some risk, especially in times of war, and hence, with the best patriotic intentions, the discussion went forward, and an account of it, with appropriate headlines, duly appeared in the daily Press.

The opening of the discussion was not altogether a happy one, as in the chairman's speech special stress was placed on the discordant voices of the numerous counsellors who pretend to give advice on the subject. Even on so elementary a matter as the number of bites to be given to each mouthful the experts, it seems, are not agreed; and though the war has silenced many controversies, here is a region in which contention still reigns. It is much the same with reference to the proper form in which food should be administered, whether in a liquid or pulpy state, or, alternatively, in a tough and fibrous fashion. Another point of controversy deals with the daily amount of proteid necessary for health and efficiency, and we are left quite uncertain whether we ought to follow the lieutenant-colonel who names 190 grammes, or should content ourselves with the 50 to 60 prescribed by the professor. There appeared to be some solid ground in the announcement that part of this proteid could be obtained from peas and beans, until a subsequent speaker remarked that such substances were apt to put a severe burden upon the processes of digestion. To find so many open fundamental questions must necessarily cause some disappointment and confusion in those who were confidently expecting authoritative guidance. Possibly some readers may have heard of conflict of opinion on such points in previous discussions, but in a debate in which the war was to be prominently presented it may well have been anticipated that these old-time issues would have been finally concluded.

A more pleasant task is to turn to the positive contributions offered by the discussion. A very definite one was to the effect that far too much jam is eaten, and this was made particularly appropriate by the added statement that as a consequence of the quantities distributed to the troops the unfortunate soldiers suffer severely from toothache, and the dentists are kept busy. Here, at least, is a doctrine quite unqualified, and of it the authorities will doubtless take due note. Another point which was presented with authority was the value of the herring, and the public were reproached for having lost their earlier love for this modest fish, but whether the war is responsible for this decline did not appear. Something, it appears, depends on the cooking, and though the remark may have been made before, it is welcome as a positive proposition. Considerable support also was afforded to the value of fats, which, instead of being made into soap and so spent upon externals, might usefully be employed as foods for the benefit of the internal economy. Cheese, too, earned a good word, and margarine was praised with moderate enthusiasm. The "richer classes" were advised to eat less meat, but the people generally were assured that a large proportion of them are habitually underfed. As a practical measure it was generally agreed that the Government, or a representative committee, should consider the whole question and should take the steps necessary in the interest of the public health and of economy. Altogether the discussion leaves the melancholy impression that the war cannot boast among its redeeming features any enlargement either of the knowledge or of the powers of our dietetic experts.

